# Human Neural Stem Cell Replacement Therapy for Amyotrophic Lateral Sclerosis by Spinal Transplantation

**DOI:** 10.1371/journal.pone.0042614

**Published:** 2012-08-20

**Authors:** Michael P. Hefferan, Jan Galik, Osamu Kakinohana, Gabriela Sekerkova, Camila Santucci, Silvia Marsala, Roman Navarro, Marian Hruska-Plochan, Karl Johe, Eva Feldman, Don W. Cleveland, Martin Marsala

**Affiliations:** 1 Neuroregeneration Laboratory, Department of Anesthesiology, University of California San Diego, La Jolla, California, United States of America; 2 Institute of Neurobiology, Slovak Academy of Sciences, Košice, Slovakia; 3 Institute of Biology and Ecology, Faculty of Science, Pavol Jozef Safarik University, Košice, Slovakia; 4 Department of Cell and Molecular Biology, Feinberg School of Medicine, Northwestern University, Chicago, Illinois, United States of America; 5 Neuralstem Inc, Rockville, Maryland, United States of America; 6 Department of Neurology, University of Michigan, Ann Arbor, Michigan, United States of America; 7 Ludwig Institute and Department of Cellular and Molecular Medicine, School of Medicine, University of California San Diego, La Jolla, California, United States of America; 8 Institute of Animal Physiology and Genetics, Academy of Sciences of the Czech Republic, Libechov, Czech Republic; 9 Department of Cell Biology, Faculty of Science, Charles University in Prague, Prague, Czech Republic; Creighton University, United States of America

## Abstract

**Background:**

Mutation in the ubiquitously expressed cytoplasmic superoxide dismutase (SOD1) causes an inherited form of Amyotrophic Lateral Sclerosis (ALS). Mutant synthesis in motor neurons drives disease onset and early disease progression. Previous experimental studies have shown that spinal grafting of human fetal spinal neural stem cells (hNSCs) into the lumbar spinal cord of SOD1^G93A^ rats leads to a moderate therapeutical effect as evidenced by local α-motoneuron sparing and extension of lifespan. The aim of the present study was to analyze the degree of therapeutical effect of hNSCs once grafted into the lumbar spinal ventral horn in presymptomatic immunosuppressed SOD1^G93A^ rats and to assess the presence and functional integrity of the descending motor system in symptomatic SOD1^G93A^ animals.

**Methods/Principal Findings:**

Presymptomatic SOD1^G93A^ rats (60–65 days old) received spinal lumbar injections of hNSCs. After cell grafting, disease onset, disease progression and lifespan were analyzed. In separate symptomatic SOD1^G93A^ rats, the presence and functional conductivity of descending motor tracts (corticospinal and rubrospinal) was analyzed by spinal surface recording electrodes after electrical stimulation of the motor cortex. Silver impregnation of lumbar spinal cord sections and descending motor axon counting in plastic spinal cord sections were used to validate morphologically the integrity of descending motor tracts. Grafting of hNSCs into the lumbar spinal cord of SOD1^G93A^ rats protected α-motoneurons in the vicinity of grafted cells, provided transient functional improvement, but offered no protection to α-motoneuron pools distant from grafted lumbar segments. Analysis of motor-evoked potentials recorded from the thoracic spinal cord of symptomatic SOD1^G93A^ rats showed a near complete loss of descending motor tract conduction, corresponding to a significant (50–65%) loss of large caliber descending motor axons.

**Conclusions/Significance:**

These data demonstrate that in order to achieve a more clinically-adequate treatment, cell-replacement/gene therapy strategies will likely require both spinal and supraspinal targets.

## Introduction

Amyotrophic lateral sclerosis (ALS), also known as Lou Gehrig's disease, is characterized by the progressive development of motor dysfunction, α-motoneuron degeneration and death, in turn producing progressive fatal paralysis. Both inherited and sporadic instances of disease combine lower α-motoneuron degeneration and upper motor neuron lesion(s) [1], [2]. Depending on the time course of α-motoneuron degeneration within spinal cord segments (cervical, lumbar or both), the early clinical manifestation of disease typically presents as motor weakness with progressive loss of ambulatory and/or respiratory function. In addition to motor deficits, several other qualitatively distinct neurological symptoms including muscle spasticity and segmental hyper-reflexia are also frequently seen during disease progression [1].

While the pathological mechanisms leading to progressive neuronal degeneration are likely multi-factorial, there is converging evidence for the role of both motor neurons and astrocytes as key disease mediators. Early studies identified functional abnormalities in astroglial-specific glutamate transporters (EAAT2) in both sporadic and familial ALS human tissues [3], as well as mutant SOD1 transgenic rodent models [4], [5]Howlan}. The role of non-motor neurons in the evolution of α-motoneuron degeneration in ALS was initially validated by analysis of chimeric mouse models that were mixtures of normal and mutant SOD1 expressing cells. Those studies revealed that normal motor neurons within an ALS-causing mutant cell environment develop disease-related damage [6]. In addition, analysis of other chimeric mice in which 100% of motor neurons expressed high levels of a disease-causing ALS mutation in SOD1 demonstrated that the presence of normal non-neuronal cells could delay or eliminate disease [7]. Diminished mutant SOD1 synthesis from astrocytes strongly slowed the rate of disease progression [7]. Finally, in vitro studies have provided evidence that ALS glia isolated from mutant SOD1 transgenic mice release factors (not yet identified) that are sufficient to trigger human and rodent motor neuron degeneration in vitro [8]–[11]. Thus, the loss of astrocyte–mediated glutamate buffering capacity and the secretion of toxic factors from local astrocytes may both contribute to neuronal degeneration in ALS.

Consistent with these mechanism-exploratory studies, which identified the role of mutated astrocytes in disease progression, recent *in vivo* spinal cell grafting data provided evidence that local segmental enrichment with wild-type neural or astrocyte precursors leads to a certain degree of neuroprotection. Focal enrichment of normal astrocytes, by transplantation of fetal rat spinal cord-derived, lineage-restricted astrocyte precursors (AP), produced significant benefit in a rat model that develops fatal motor neuron disease from expression of mutant SOD1^G93A^. AP transplantation adjacent to cervical spinal cord respiratory motor neuron pools, the principal cells whose dysfunction leads to death in ALS, survived in diseased tissue, differentiated efficiently into astrocytes and reduced microgliosis in the cervical spinal cords of SOD1^G93A^ rats. Functionally, AP-grafted animals showed: i) extended survival and disease duration, ii) attenuated motor neuron loss, iii) slowed declines in forelimb motor performance, and iv) improved respiratory functions. It was hypothesized that neuroprotection was mediated in part by the primary astrocyte glutamate transporter EAAT2 expressed in grafted cells (called GLT1 in rodents) [12].

Mutant damage within motor neurons has also been demonstrated to play a central role in development of disease. In rodent models, diminishing mutant SOD1 synthesis within motor neurons (by selective transgene inactivation [7], [13] or viral-mediated siRNA delivered by retrograde transport after intramuscular injection [14], [15] can sharply delay disease onset. Spinal lumbar grafting of human fetal spinal neural stem cells (i.e., the same cells as used in our current study) in immunosuppressed SOD1^G93A^ rats has been reported to yield long-term graft survival (average 86 days) and formation of synapses with the host neurons. Grafted animals were reported to have disease onset delayed by 7 days and the age at which limb paralysis was reached was extended by 11 days [16], [17].

The compelling evidence of non-cell autonomous contributions to disease in models of SOD1 mediated ALS makes cell replacement therapy an attractive option. We now report the long term survival and differentiation into neurons and astrocytes of human fetal spinal neural stem cells (hNSCs) after grafting into the vicinity of lumbar spinal α-motoneurons and a local transient functional benefit after grafting into immunosuppressed presymptomatic SOD1^G93A^ rats.

## Results

### Grafted human fetal spinal neural stem cells (hNSCs) show long term survival, develop neuronal morophologies and form synapses with host α-motoneurons

**Figure 1:**
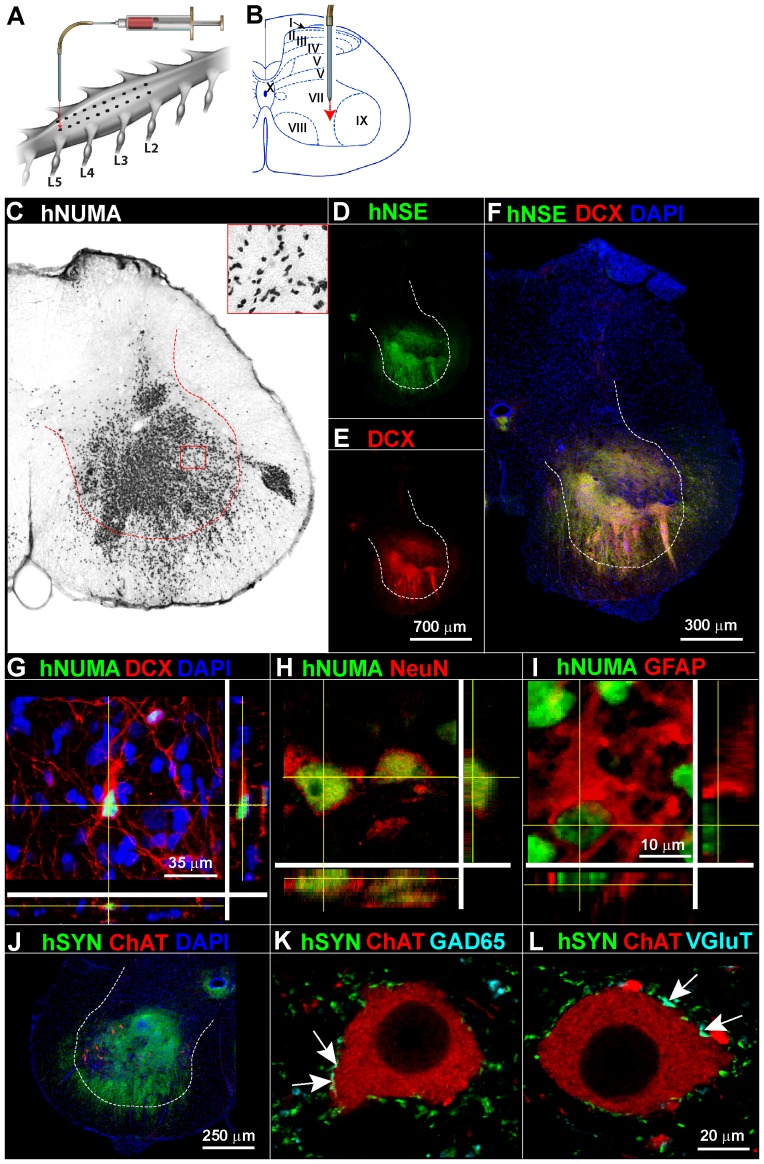
Human spinal neural stem cells grafted into lumbar spinal cord of SOD1^G93A^ rats show long term survival and preferential neuronal differentiation. Using a glass capillary SOD1^G93A^ rats (60–65 days old) received 10 bilateral injections of hNSCs targeted into the intermediate zone (lamina VII) and ventral horn (lamina VIII, IX) of L2–L5 spinal segments (A, B). Lumbar spinal cord sections from cell-grafted SOD1^G93A^ animals immunostained for human nuclear matrix antigen (hNUMA) to identify all cells of human origin and developed by diaminobenzidine (C) shows a dense population of nucleus-like structures throughout the mid- and deep laminae (dashed line delineates the ventral horn) as well as more disperse nuclei outside the graft core and often into the white matter (inset). Neighboring sections revealed cell grafts that were strongly immunoreactive (IR) for human neuron-specific enolase (hNSE) and doublecortin (DCX; D–G). Other hNUMA-IR cells were found to react with the neuronal nuclear protein NeuN (H) and glial fibrillary acidic protein (GFAP; I). Human synaptophysin (hSYN) was detected throughout the cell grafts and was often found in the vicinity of persisting motoneurons (ChAT; choline acetyltranseferase) in lamina IX and extending into the adjacent white matter (J). Single optical layer confocal images of surviving α -motoneurons show hSYN-IR bouton-like structures adjacent to the outer membrane of the soma (K, L). Only occasional hSYN-IR boutons co-stained with the GABA-ergic cell marker glutamate decarboxylase (GAD65; K); glutamatergic boutons were located by identifying specific glutamate vesicular transporters 1/2/3 (VGluT) and similarly showed only rare boutons also reactive for hSYN (L). Arrows show some examples of double-immunoreactive structures. Scale bar: 300 µm (C, F), 700 µm (D, E), 35 µm (G), 10 µm (H, I), 250 µm (J), 20 µm (K, L).

Twenty-four, SOD1^G93A^ rats (12 male, 12 female; 60–65 days old) received 10 bilateral injections of hNSCs targeted into ventral horn of L2–L5 spinal segments (**Fig. 1A, B**). Sixteen additional animals received media only. All animals survived until endstage disease, with the exception of one media- and two cell-treated animals which died perioperatively. Immunohistological examination of spinal cord tissue from animals at endstage using an antibody recognizing the human, but not rat, nuclear matrix protein (hNUMA) revealed identifiable human grafts in 18/22 animals. Four animals were graft-negative as indicated by injection tracks which were clearly visible, but with no human antigen detected (data not shown). hNUMA-immunoreactive cell grafts were found in the central and deep gray matter (laminae VII–IX) and sometimes extended into white matter (**Fig. 1C**). The overall appearance of spinal cords from cell-grafted animals was generally unremarkable, with only some examples of cell grafts extended into the white matter and slight enlargement of the spinal cord was then sometimes noted typically in the area closest to the graft. The pattern of engraftment identified by antibodies selective for human neuron-specific enolase (hNSE) (**Fig. 1D**) or doublecortin (DCX; **Fig. 1E**), an early postmitotic neuronal marker, closely matched that seen with hNUMA. Essentially all structures within the graft core labeled for both hNSE and doublecortin (**Fig. 1F**). Numerous individual doublecortin/hNUMA-immunopositive cells were readily found outside the graft core and had long neural-like processes with axonal varicosities (often more than 500 µm in length; **Fig. 1G**).

Quantitative analyses showed that 78±6% of hNUMA-positive nuclei were surrounded by a doublecortin-positive cytoskeleton, suggesting that those cells were young, migrating, post-mitotic neurons. Likewise, no cells with doublecortin were found lacking an hNUMA-positive nucleus. Similar to the hNSE staining pattern, fibers with doublecortin were found extending >500 µm radially from the graft core, sometimes crossing through/near lamina X to the opposite side. On average 12.5±1.2% of hNUMA-positive cells expressed the mature neuron marker NeuN (**Fig. 1H**). Graft cores identified with hNUMA were also intensely stained for the neuron-specific cytoskeletal protein βIII tubulin (TUJ1; [18]; **Fig. S1A–C**) and GAP43 (**Fig. S1D–G**). Only 1.7±1.1% of hNUMA-positive cells appeared to be astrocytes with detectable GFAP filaments (**Fig. 1I**). Only 3.2±0.9% of hNUMA-positive cells reacted with the mature oligodendrocyte marker APC (not shown).

Examination of grafted spinal cord tissue revealed a dense fiber-like pattern of human-specific synaptophysin within the core and extending outward into the adjacent tissue (**Fig. 1J** **, Fig. S2A–D**). Persisting host motoneurons (α and γ) were frequently found to have human-specific synaptophysin-positive bouton-like structures adjacent to their cell body and associated processes. By examining specific neurotransmitter phenotype markers in 0.5 µm-thick optical sections, 0.8±0.3% of human synaptophysin-positive structures were co-labeled with glutamate decarboxylase 65 (GAD65; **Fig. 1K**; **Fig. S2E–H**), a marker for γ-aminobutyric acid-producing neurons. Immunostaining for each of the three vesicular glutamate transporters known to exist in the spinal cord (VGluT 1, 2, and 3) revealed that 1.3±0.5% of boutons with human synaptophysin were glutamatergic (**Fig. 1L**; **Fig. S2I–L**). Glycinergic boutons, identified by immunostaining for a glycine transporter (GlyT2) represented 0.9±0.6% of these boutons (**Fig. S2M–P**), consistent with a human stem cell-derived glycinergic neuron. No synaptophysin-positive structures were found to co-label with the motor neuron marker ChAT (**Fig. 1K, L**; **Fig. S2H, L, P**). Indeed, in the vast majority (>97%) of cases human synaptophysin-positive cells did not contain any differentiated cell marker, indicating a persistent immature phenotype.

### Human spinal neural stem cells protect α-motoneurons in grafted segments

**Figure 2:**
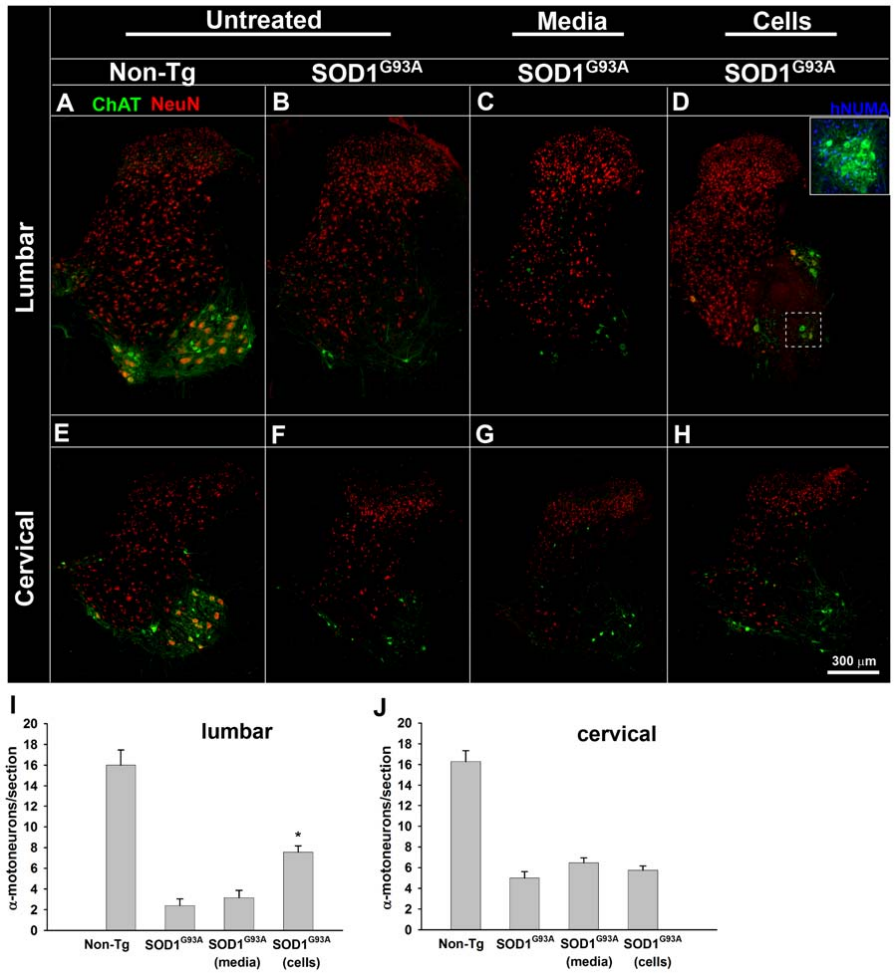
SOD1^G93A^ rats receiving lumbar grafts of human spinal neural stem cells show higher α-motoneuron survival at the lumbar but not cervical spinal segments. Lumbar transverse spinal cord sections from untreated, non-transgenic (Non-Tg; A) and untreated SOD1^G93A^ transgenic (SOD1^G93A^; B) animals, as well as SOD1^G93A^ animals treated with media (SOD1^G93A^ media; C) or human spinal neural stem cell grafts (SOD1^G93A^ cells, D) were double immunostained to identify all mature neurons (NeuN) and cholinergic cells (α-motoneurons, ChAT). While a dramatic reduction in ChAT-IR cells was noted in all SOD1^G93A^ animals, regardless of treatment, quantitative analysis (I) of persisting α-motoneurons showed a significant increase in the α-motoneuron pool in the cell-grafted animals (D, I). Using adjacent histological sections, inset (D) shows an example of a group of transplanted cells (positive for human nuclear matrix antigen; hNUMA) near a pool of surviving α-motoneurons. Sections taken from the same animals but from the cervical level (C5/6) also show a dramatic reduction in the motoneuron pool but with no apparent protective effect afforded by the lumbar cell grafts (E–H, J). Scale bar: 300 µm. * P<0.001 compared to non-transgenic, P = 0.03 compared to the media-treated group; one-way ANOVA.

In order to assess a possible protective effect of grafted hNSCs on lumbar α-motorneuron survival we performed quantitative histological analyses of persisting lamina IX α-motoneurons in normal, SOD1^G93A^, SOD1^G93A^/hNSCs-grafted, and SOD1^G93A^/media-treated animals in the lumbar (L4–L5) spinal cord segments. Representative half spinal cord images from each animal group are shown in **Fig. 2A–D**. In untreated SOD1^G93A^ animals, there was an average 85±7% reduction in the number of α-motoneurons per histological section compared to untreated, non-transgenic rats (**Fig. 2I**). While media-treated SOD1^G93A^ rats showed an 80±9% reduction in the α-motoneuron population, in hNSCs-grafted animals the lateral α-motorneuron pool showed only a 53±5% decrease relative to normal animals), which was a significantly smaller reduction than the untreated or media-treated SOD1^G93A^ animal groups (P<0.001; one-way ANOVA).

Analysis of α-motoneuron survival in the phrenic nucleus (C5–6) was used to test whether the lumbar cell grafts provided protection in cervical motoneuron pools distant from grafted lumbar segments. Half spinal cord images from untreated normal animals showed an intensely-stained (choline acetyl transferase - ChAT) motoneuron pool in cervical spinal cord (**Fig. 2E**). Untreated SOD1^G93A^ animals at endstage disease developed a dramatic loss in the α-motoneuron population (**Fig. 2F, J**; a 69±4% reduction compared to age-matched non-transgenic littermates). Media-treated (**Fig. 2G**) and hNSCs-grafted (**Fig. 2H**) SOD1^G93A^ animals had similarly-reduced cervical motor pools (60±4% and 65±4% of normal littermates, respectively).

### Grafted human spinal neural stem cells ameliorate lumbar astrogliosis and microglial activation

To test how engraftment of hNSCs affects the astroglial and microglial activation that prominently develop in SOD1^G93A^ rats, region-specific (lamina IX) astrogliosis and microglial activation was assessed by densitometric analysis of glial fibrillary acidic protein (GFAP) and ionized calcium-binding adaptor molecule 1 (Iba1) immunoreactivity, respectively. Untreated normal rats showed minimal GFAP reactivity as expected for wild-type animals, with very thin processes in astrocytes and usually no discernable cell body (**Fig. S3A**). Iba1 immunoreactivity was also comparable to that of wild-type naïve animals, with microglia with long thin processes (**Fig. S3B**). However, by endstage disease, intense reactive astrogliosis (i.e., hypertrophic astrocytes with short, thick processes and enlarged soma) was found in the untreated SOD1^G93A^ rats (**Fig. S3D, M**). Microglial activation in SOD1^G93A^ mutant animals paralleled the reactive astrogliosis with a significant increase in the presence of Iba1+ cells throughout the ventral horn (and concentrated in lamina VIII and IX - **Fig. S3E, M**). While media treatment did not alter development of GFAP or Iba1 reactivity in laminae IX (compared to untreated, mutant SOD1^G93A^ tissue; **Fig. S3I, M**), hNSCs-transplantation reduced the overall immunoreactivity for GFAP and Iba1 (**Fig. S3L, M**). While media-treated tissue had GFAP and Iba1 reactivities 8±0.9 and 6±0.8 fold greater than that in normal animals, both were reduced [to 4±0.8 fold (p = 0.04; one-way ANOVA) and 3.6±0.4 fold (p = 0.02; one-way ANOVA)] in hNSCs grafted animals, respectively.

### Grafted human spinal neural stem cells migrate extensively and form synapses

**Figure 3:**
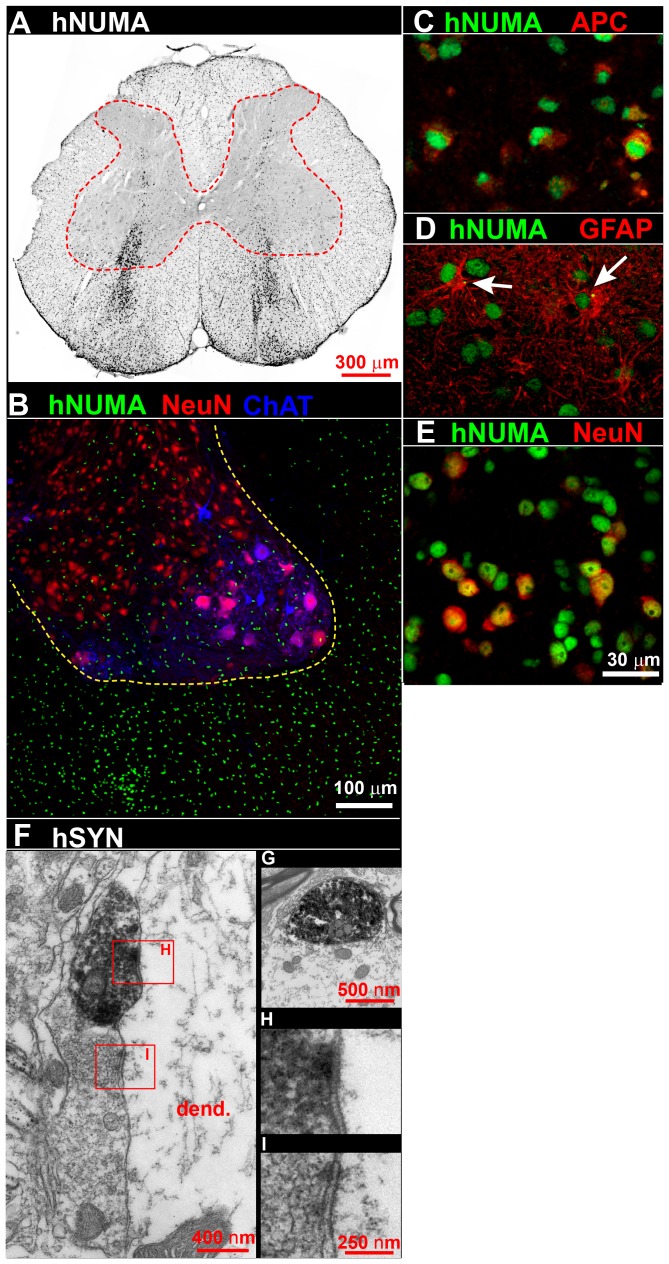
Effective re-population of lumbar gray and white matter by human spinal neural stem cells nine months after lumbar transplantation in immunodeficient rats. When human spinal neural stem cells (same cell line as were used in the grafting experiments in **Fig. 1**, **2** and **Fig. S2, S3**) were transplanted into the lumbar spinal cord of immunodeficient rats, histological sections taken after nine months of survival revealed a near homogenous distribution of human cells (hNUMA; A, B) in both gray and white matter. In white matter hNUMA-IR cells were primarily oligodendrocytes (adenomatous polyposis coli; APC; C) or astrocytes (GFAP; D) while in gray matter a neuronal phenotype (NeuN; E) was identified. Electron microscopy revealed axon terminals, enriched with human synaptophysin immunoreaction product (F, G), forming synapses with host neurons or dendrites (F, H, I). Scale bar: 300 µm (A), 100 µm (B), 30 µm (C–E), 400 nm (F), 500 nm (G), 250 nm (H, I).

To assess the degree of cell migration and synapse formation at extended periods after grafting, we compared non-quantitatively the distribution of grafted (hNUMA+) cells 9 months after transplantation into lumbar spinal cords of immunodeficient rats with the pattern seen in cell-grafted SOD1^G93A^ rats (i.e., around 78 days after cell transplantation). Extensive migration of grafted cells was seen into both white and gray mater (**Fig. 3A, B** **; Fig. S4A,B**). Double staining of hNUMA+ sections showed that the hNSCs-derived cells localized in white matter acquired only glial phenotypes (**Fig. 3C, D**) [as shown by co-staining with antibodies to neuronal (NeuN, human neural specific enolase - NSE, CHAT) or non-neuronal (GFAP and the mature oligodendrocyte cell marker APC) proteins]. hNSCs-derived cells in the gray matter, on the other hand, had both neuronal and non-neuronal markers (**Fig. 3E**). Electron microscopy confirmed that human synaptophysin (hSYN) immunoreactive axonal terminals had formed synapses with adjacent host neuron-derived dendrites (**Fig. 3F–I**).

### Near complete loss of spinally-recorded motor-evoked potentials in non-treated SOD1^G93A^ animals

**Figure 4:**
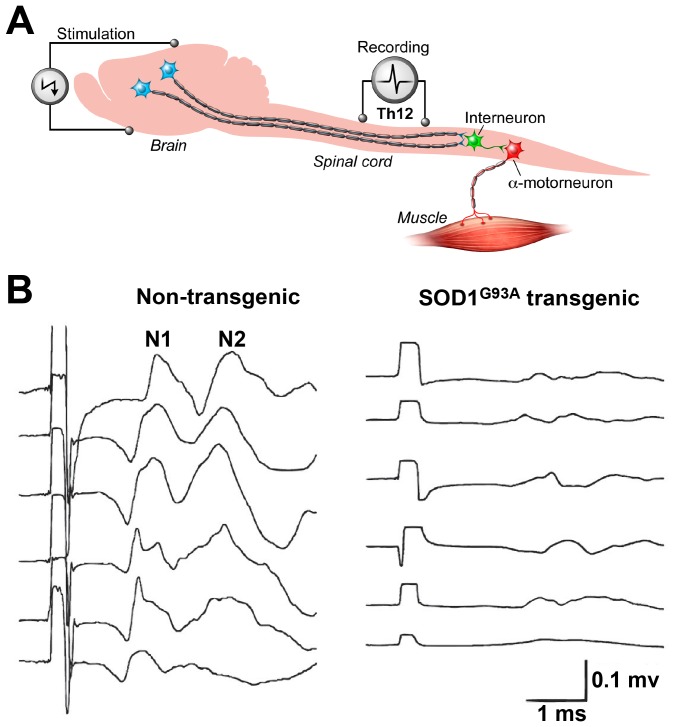
Motor-evoked potentials recorded from the dorsal surface of T12 spinal cord are near completely lost in endstage SOD1^G93A^ rats. To identify the conductivity of descending motor axons MEPs were elicited by electrical stimulation of motor cortex and extra-pyramidal system and responses recorded from exposed T12 spinal segments (A). Recordings in 6 non-transgenic animals showed consistent compound action potentials consisting of N1 and N2 waves with an average latencies of 1.6–2.8 ms respectively (B). In contrast, recording in 6 transgenic SOD1^G93A^ endstage animals showed near complete loss of N1 and N2 waves.

In order to assess the upper motor neuron connectivity at the spinal level, motor-evoked potentials were recorded in a separate group of endstage SOD1^G93A^ rats with no previous manipulations (i.e. no spinal media or cell injections) and age-matched non-transgenic control animals. Motor-evoked potentials (MEPs) were recorded from the dorsal surface of exposed thoracic T12 segment after electrical stimulation of the motor cortex (**Fig. 4A**). MEPs consist of multiple waves, with the two earliest peaks, N1 and N2, corresponding to the activation of extrapyramidal system [19]–[21]. In non-transgenic animals, the N1 wave (average amplitude 140.7±39.0 mV; **Table 1**) was recorded at 1.6±0.2 ms and the N2 wave (average amplitude 88.4±51.3 mV) 2.8±0.3 ms after the stimulus, yielding calculated conduction velocities of 52–62 and 28–32 m/s, respectively. In SOD1^G93A^ endstage animals, the N1 amplitude was reduced to 1/6^th^ the normal level (25.5±6.4 mV, p<0.05 compared to normal animals; t-test) and latency was increased by 55% (to 2.5±0.2 ms; p<0.05; t-test) (**Fig. 4 B** **,** **Table 1**). Similarly, N2 amplitude was reduced to about 30% of normal (to 25.1±9.1 mV) and latency was increased 25% (to 3.5±0.5 ms, p<0.05; t-test) (**Fig. 4B** **,** **Table 1**).

**Table 1:** Analysis of motor-evoked potentials recorded from the dorsal surface of the T12 spinal cord segment in non-transgenic and endstage transgenic SDO1^G93A^ rats.

	Amplitude (mV ± SD)		Latency (ms ± SD)	
	N1	N2	N1	N2
**Non-Tg**	140.7±39.0	88.4±51.3	1.6±0.2	2.8±0.3
**SOD1^G93A^**	25.5±6.4*	25.1±9.1*	2.5±0.2*	3.5±0.5*

The amplitudes and latencies of the N1 and N2 waves were calculated and averaged from 6 non-tg and 6 SOD1^G93A^ endstage animals. (* P<0.05).

### Loss of large, descending myelinated axons in spinal white matter of mutant SOD1^G93A^

**Figure 5:**
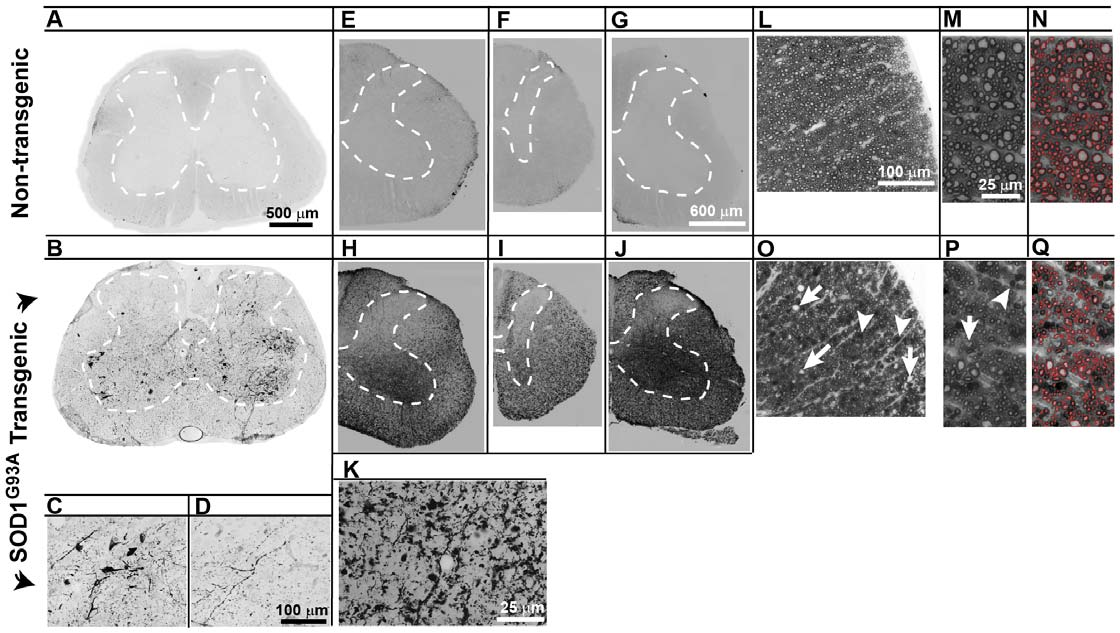
Significant degeneration of descending medium-, and large-size myelinated axons in lumbar segments in endstage SOD^G93A^ rats. Using the Gallyas silver impregnation technique, degenerating neurons were detected mostly within the ventral horn, although reactive cells were often noted in upper lamina (A- non-transgenic, B- SOD1^G93A^endstage). Closer examination shows fragmented cells and fibers not only within the grey matter, but also in the adjacent white matter (C, D). The de Olmos modified cupric-silver stain was used to help detect degenerating axons and dendrites; non-transgenic tissue showed no appreciable staining (E – cervical, F-thoracic, G – lumbar), while SOD1^G93A^ tissue reacted intensely, not only in the ventral horns but throughout the gray matter, sparing only lamina I–III (H–J). Closer examination of the white matter showed argyrophilic punctate structures corresponding to silver deposits in disintegrated axons (K). Semithin (1 µm) plastic-embedded tissue sections were used to quantify changes in the axonal population in the lateral and ventral columns. While clearly-delineated axons of varied caliber were readily-outlined by the osmium-stained myelin in non-transgenic animals (L–N), tissue from endstage SOD1^G93A^ animals had an easily recognizable reduction of large caliber myelinated axons (O–Q). Lumbar white matter in SOD1^G93A^ endstage animals also contained numerous vacuoles (O, P, arrows) and frequent osmium-dense deposits (O, P, arrowheads). Scale bar: 500 µm (A, B), 100 µm (C, D), 600 µm (E–J), 25 µm (K), 100 µm(L, O), 25 µm (M, N, P, Q).

Histological analysis of the integrity of descending motor tract degeneration in cervical and lumbar spinal cord was determined in terminal SOD1^G93A^ rats which were previously used for MEPs recording. Silver impregnation techniques were employed to detect the presence and distribution of axo-dendritic disintegration, which is marked by silver deposition [22]–[25]. While lumbar sections from the normal animals had minimal silver deposition (**Fig. 5A**), similar sections from SOD1^G93A^ animals had numerous degenerating α-motoneurons and interneurons that stained intensely (**Fig. 5B, C**) and an abundant number of fragmented fibers coursing throughout laminae IV–X (**Fig. 5D**). Numerous argyrophilic punctate structures were also prominent in most white matter regions, particularly in the lateral and ventral columns. Using a less sensitive silver method (de Olmos aminocupric silver impregnation), massive axo-dendritic degeneration (compare the tissue from normal animals [**Fig. 5E–G**] with that from SOD1^G93A^ animals [**Fig. 5H–K**]) was identified in several areas in the cervical, thoracic and lumbar spinal cord segments including: i) the lateral and ventral white matter, ii) laminae IV–IX within the gray matter, and iii) to a lesser degree in the dorsal columns (**Fig. 5H–K**).

The total number of remaining axons and their calibers (0.5–2, 2–5, 5–14 µm in diameter) were determined in the lateral and ventral funiculi [using semi-automated image analysis of osmium-treated 1 µm plastic sections taken from lumbar spinal cord of normal (n = 2; **Fig. 5 L–N**) and SOD1^G93A^ (n = 2) endstage animals with no previous manipulations (i.e. no spinal media or cell injections; **Fig. 5 O–Q**)]. In normal tissue, axons of varied caliber were clearly-outlined with myelin (**Fig. 5M, N**). On the other hand, an easily recognizable reduction in the number of large caliber myelinated axons was found in endstage tissue from SOD1^G93A^ animals (compare **Fig. 5 M, N** to **P, Q**). Axonal loss in the lateral funiculus averaged 19% for 0.5–2 µm caliber axons, 40% for 2–5 µm caliber axons, and 57% of axons 5–14 µm in diameter (**Table 2**). Numerous medium sized (10–15 µm) and large (15–25 µm) vacuoles, likely evolving at sites of previous axonal degeneration and phagocytic activity (**Fig. 5 O, P**, arrows), were prominent in lumbar white matter of the SOD1^G93A^ animals. In addition, frequent osmium-dense deposits that were likely macrophages and activated microglia were consistently identified (**Fig. 5O, P**, arrowheads).

**Table 2:** Quantification of lateral and ventral funiculi axons in lumbar spinal cord segments of non-transgenic and endstage transgenic SOD^G93A^ rats.

Lateral Funiculus				
	Total axons	0.5–2.0 µm	2.0–5.0 µm	5.0–14 µm
**Non-Tg**	45681	31982	11529	2079
**Non-Tg**	62656	43444	16782	2299
**SOD1^G93A^**	40422	30943	8411	994
**SOD1^G93A^**	39729	30315	8467	881

Ventral Funiculus				
	Total axons	0.5–2.0 µm	2.0–5.0 µm	5.0–14 µm
**Non-Tg**	14330	8764	4433	1087
**Non-Tg**	16500	9830	5382	1247
**SOD1^G93A^**	11532	8037	2984	489
**SOD1^G93A^**	14002	10285	3153	536

Quantification of the axonal population in the lateral and ventral columns showed an easily recognizable reduction of large caliber myelinated axons.

### Transient retention in neurologic and reflex activity from grafting of human spinal neural stem cells, but no effect on survival of SOD1^G93A^ animals

**Figure 6:**
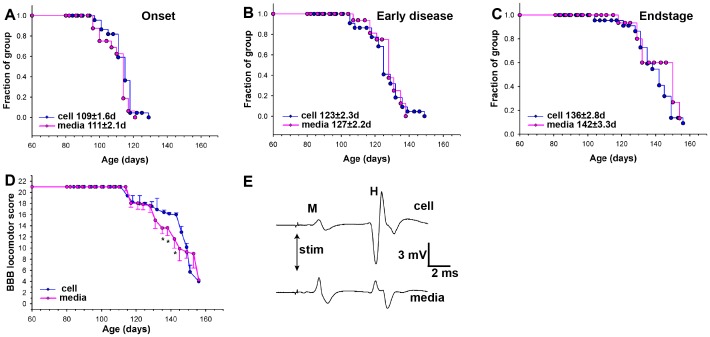
SOD1^G93A^ rats receiving lumbar grafts of human spinal neural stem cells showed transient protection of hindlimb motor function and Hoffmann reflex. Population analyses of media-treated and cell-grafted animals showed no difference in: disease onset - defined as peak body weight (A), early disease progression - defined as the age at which animals had lost 10% body weight (B), or survival (C). Hindlimb motor function assessed by BBB neurological score (D) showed a significantly better score in hNSCs-grafted animals between ages 135–142 d. Similarly, Hoffman reflex recorded in cell-grafted animals during that time period had significantly higher average amplitudes than those from the media-treated group (E), (* P<0.05).

Development and progression of ALS-like disease in SOD1^G93A^ was followed after lumbar grafting of hNSCs of presmyptomatic (60–65 days old) SOD1^G93A^ rats (**Fig. 1A**). Disease onset, defined as the peak animal body weight (**Fig. 6A**), in grafted animals was not different from untreated animals (109±2d, 111±2, and 108±3 for grafted animals, media-injected and untreated SOD1^G93A^ animals; p = 0.41; t-test). Grafting also produced no statistically significant effect on progression to an early disease point (identified by the age at which the animal had lost 10% of its body weight from denervation-induced muscle atrophy; **Fig. 6B**) between hNSCs-grafted animals and those that received media only (123±2d versus 127±2d, respectively; P = 0.122; t-test). Overall survival was also not significantly affected between the hNSCs-grafted or media-injected animals (136±3d and 142±3d, p = 0.18; t-test) (**Fig. 6C**). Analyzing males and females separately failed to reveal any significant difference in any disease index (data not shown).

On the other hand, neurological function (using a well-established BBB scoring scale measured from each animal every 3–4 days) was significantly preserved at age 135–142d in the cell-grafted animals compared with the media-treated group (**Fig. 6D**; p<0.05; t-test). Similarly, at the same age (**Fig. 6E**) recording of Hoffmann reflex revealed a higher average H-wave amplitude for the hNSCs-grafted group compared to the media-treated animals (3.5±0.6 vs. 1.1±0.3 mV; p<0.05; t-test), indicating functional preservation between sensory Ia afferent, α-motoneuron and motor plate.

## Discussion

Our results show that human fetal spinal neural stem cells (hNSCs) grafted into the lumbar ventral horn in SOD1^G93A^ rats provides a region-specific neuroprotective effect in the vicinity of the grafted cells, including a higher number of surviving α-motoneurons, transient improvement in ambulatory function, corresponding transient preservation of H-reflex activity, and decreased inflammatory responses. Our findings add to earlier evidence that reported i) preservation of α-motoneurons in lumbar spinal cord in SOD1^G93A^ rats after unilateral grafting of human neural stem cells genetically modified to produce glial cell line-derived neurotrophic factor (GDNF) [26] and ii) functional improvement (as assessed by extended survival and disease duration, improved forelimb motor and respiratory functions) and corresponding decrease in α-motoneuron degeneration in SOD1^G93A^ rats receiving cervical grafts of rat astrocyte precursors [12].

Despite initial reports 18 and 9 years ago, respectively, of SOD1^G93A^ mutant mouse and rat transgenic models [5], [27], there is only one characterization of the degree of upper motor neuron degeneration in one mouse model [28] and none in the rat. To this, our current MEPs data show that in symptomatic animals there is a near complete loss of descending motor tract conductivity measured directly from the dural surface of the exposed Th12 spinal segment (**Fig. 4**). This functional loss corresponds with massive axonal degeneration in the white matter particularly affecting large caliber myelinated axons in the anterior and lateral funiculus (**Fig. 5**). This is consistent with a classical picture of spinal histopathological changes in ALS patients which, in addition to spinal α-motoneuronal loss, is characterized by upper motor neuron lesion and degeneration involving the entire corticospinal tract [29]–[32]. Interestingly, in contrast to humans, preferential degeneration of axons in lateral and ventral funiculus was seen in symptomatic SOD1^G93A^ rats while relatively spared axonal populations were seen in the dorsal funiculus (i.e., the region of corticospinal tract in rats). Whether or not it is the result of species specificity is not known, however, one important functional distinction between the organization of the different motor systems involved in voluntary motor function exists between human and rodents. It has been demonstrated that after complete transection of the pyramidal tract in rats a considerable amount of motor function persists [33]. It is believed that the activity of the extrapyramidal system (such as rubrospinal tract which descend in lateral funiculi in the rat) plays a more dominant role in the initiation and maintenance of voluntary movement in rats. We speculate that a more pronounced axonal loss seen in the lateral funiculi in symptomatic SOD1^G93A^ rats can thus be attributed to a specific motor circuitry-controlling function of descending motor axons residing in this region.

Based on these histopathological and functional data demonstrating an extensive loss of descending motor system in SOD1^G93A^ rats it is readily apparent that unless the functional integrity of all components of the motor neuraxis is maintained (or restored) by a given treatment, only local and/or time-limited functional protection can be achieved, just what we have produced with hNSCs grafts.

We found no survival benefit versus our control media-injected group, despite transient local improvement. This was not unexpected, since for humane reasons survival in this animal model is defined by a loss of righting reflex (i.e., the ability of the animal to right itself). An intact righting reflex requires coordinated hindlimb and forelimb motor function and continuing functional coupling of the upper and lower motor neuron system. In deficits which include upper and lower motor neuron degeneration (such as seen in SOD1^G93A^ rats), region-restricted treatments (as achieved after spinal segmental cell grafting) is not expected to significantly modify upper motor neuron degeneration and loss and the associated progressive decline in righting reflex. Nevertheless, using lumbar spinal grafting of human spinal neural stem cells similar to those of our current study, Xu et al. (2006) [16] previously reported an apparent lifespan extension of SOD1^G93A^ rats of 11 days (average 149 days) compared to control animals receiving injection of dead cells (average 138 days). Similar to our study a significantly higher number of persisting lumbar α-motoneurons was found in treated animals. More recently, Xu et al. (2011) reported a lifespan extension of SOD1^G93A^ rats by 17 days after dual cervical (C4–C5) and lumbar (L4–L5) transplantation of the same human spinal neural stem cell line as used in our current study [34]. Given the robust graft survival, cell differentiation and migration seen in our study, we speculate that the differences between ours and these prior studies may reflect the occurrence of natural drift in the onset of disease between different cohorts of animals (a feature that has been argued to necessitate >25 animals per group in order to draw statistically valid conclusions) [35] and/or difference in the design of the control groups (i.e., injections of dead cells vs. media only) and potentiation of local neuronal degeneration in dead cell-injected animals.

Several differential characteristics between rodent models and human ALS patients need to be considered when predicting the potential value of spinal cell replacement therapies in human patients. First is a significantly different time course of disease progression in rodents and human patients. In SOD1^G93A^ rodent models the average duration of disease from the initiation of denervation-induced weight loss to terminal stage is 60–70 days in mice [5], [27], [36], [37] and is around 27 days in rat [38]. In human ALS patients it can range between months to several years [1], [32]. Such differences have a fundamental impact on the degree of engraftment, maturation and migration of grafted cells, and can ultimately define the degree of expected neuroprotection. In our current experimental design, SOD1^G93A^ rats were implanted at age ∼65 days (i.e., on average ∼44 days prior to disease onset) and had an average survival time of 75 days after cell grafting (an average of ∼140 days to endstage). While robust neuronal differentiation was noted in grafted animals, only limited cell migration was seen in the short term to areas distant from cell-injected regions. Cell migration at 9 months post-grafting in naive, immunodeficient rats, a time period comparable to that expected in human patients receiving spinal grafts, was determined. In contrast to short times post-grafting, much more robust cell migration was seen, with a homogenous distribution of grafted cells identified in white and gray matter (**Fig. 3**). Cells found in white matter showed near-exclusive differentiation towards astrocytes and oligodendrocytes. These findings support the likelihood that comparable spinal cell repopulation can be expected in human patients postgrafting and that this can be associated with a more relevant functional-protective effect. Combined cell grafting strategies targeted in parallel to spinal and supraspinal motor centers can be anticipated, therefore, to provide a substantial degree of neuroprotection measured both behaviorally as well as by using combined motor and somatosensory-evoked recording.

A second important component in achieving optimal therapeutic benefit in cell replacement therapies is the selection of cell lines (e.g., neural, neuronal or glial-restricted) to be used for spinal/supraspinal grafting. The primary selection criteria should reflect the disease stage and targeted cell population to be replaced, that is, cells whose damage drives early or late disease phases. Since one of the well documented mechanisms contributing to disease progression is the release of toxic factors from SOD1 mutant astrocytes and resultant neuronal degeneration [8], [9], [11], [39], the use of wild type astrocyte precursors for grafting may appear to be an attractive choice. Data from the Maragakis' group [12] demonstrated a region specific therapeutic effect when cells were grafted into the cervical gray matter in presymptomatic SOD1^G93A^ rats.

However, the absolute number of spinal (and also brain) astrocytes may not be static and there is a continued proliferation of glial precursors in the intact adult CNS [40]. In addition, there is increased astrocyte proliferation after injury such as spinal trauma or focal brain ischemia [41], [42]. More recent studies using *in vivo* BrdU incorporation assay have shown that, while the primary cellular contribution to spinal cord gliosis seen in symptomatic and endstage SOD1mutant (G93A) mice is derived from oligodendrocyte-committed NG2+ precursors (up to 49–55%), between 4–5% of BrdU-labeled cells are GFAP-immunoreactive astrocytes [43], [44]. These data show modest but continuing proliferation of mutated SOD1 astrocytes throughout the disease progression. Thus, there may be a competitive interaction between replication of the host mutated astrocytes and grafted wild-type astrocyte precursors.

Moreover, from a clinical perspective, one of the limitations in using a lineage restricted precursor population is the potential that it may be targeted for damage and potentially replaced by endogenous mutant astrocyte population. In experimental rodent studies, cells are typically grafted at pre-symptomatic stages while the clinical patient population will primarily be composed of symptomatic patients, with existing upper and/or lower motoneuron/interneuron degeneration. Because there is no evidence of neurogenesis in adult naive or trauma injured spinal cord [40], [45], the primary goal in cell replacement therapies that employ glial-restricted precursors will therefore be to stabilize or slow neural degeneration. Second, given the large number of astrocytes compared to neurons in the adult CNS, there is a limited likelihood of achieving significant astrocyte repopulation using cells lines with little or no mitotic/migratory activity, so an important characteristic of astrocyte-restricted lines for grafting is their continuing proliferative/migratory capacity after transplantation. Our current data using 9 month surviving cell-grafted immunodeficient rats demonstrate widespread grafted cell migration with no detectable tumor formation. More recently, we have observed comparable cell migration and safety of human embryonic stem cell (H9)-derived neural stem cells at 5–6 months after spinal grafting in immunodeficient rats (unpublished observations). Nonetheless, a detailed long-term safety/tumorigenicity profile will need to be established for each individual cell line before clinical use.

Finally, our evidence highlights that the use of human fetal spinal cord-derived neural stem cells which can generate astrocytes but mostly neurons *in vivo* have a limited, local therapeutic effect. Therefore, an optimal cell line will likely produce a mixture of glial and neuronal stem cells having both proliferative (at least for the astrocyte-restricted subpopulation) and migratory properties. As demonstrated in our current study, human fetal spinal cord-derived neural stem cells have such properties to a certain extent, albeit a higher ratio of astrocyte precursors in the grafted cell population might be preferable. It now remains to be determined whether human embryonic stem cells-derived neural stem cells will show a distinct differentiation ratio and more rapid migration in an ALS environment after *in vivo* grafting in humans.

In any event, cell replacement therapies should target both spinal and supraspinal components of motor neuraxis. Because the multi-site delivery of therapeutic cells is clearly technically more challenging, additional safety data as well as the development of less invasive cell delivery techniques will be required before this potentially more effective multi-site cell delivery treatment approach can successfully be introduced into clinical practice.

## Methods

### Ethics Statement and Institutional Animal Care and Use Committee approvals

This study was approved by the University of California, San Diego (UCSD) Internal Review Board (IRB), approval ID#101323.

All animal studies were carried out under protocols approved by the Institutional Animal Care and Use Committee at University of California (approval Ids # S01193 and S07016) San Diego and were in compliance with *The Association for Assessment of Laboratory Animal Care* guidelines for animal use. All studies were performed in such a manner as to minimize group size and animal suffering.

### Derivation of the human fetal spinal neural stem cells (hNSCs)

Human fetal spinal neural stem cells (hNSCs) were prepared from the cervical–upper thoracic region of spinal cord tissue obtained from a single 8-week human fetus after an elective abortion. The fetal tissue was donated by the mother in a manner fully compliant with the guidelines of NIH and FDA and approved by an outside independent review board. The spinal cord tissue was removed of meninges and dorsal root ganglia and dissociated into a single cell suspension by mechanical trituration in serum-free, modified N2 media. The modified N2 media was composed of: 100 mg/l human plasma apo-transferrin, 25 mg/l recombinant human insulin, 1.56 g/l glucose, 20 nM progesterone, 100 µM putrescine, and 30 nM sodium selenite in DMEM/F12. For growth of the hNSCs, 10 ng/ml bFGF as the sole mitogen was added to the modified N2 media (growth media). The initial culture was serially expanded as a monolayer culture in precoated flasks (T-175) or plates [46]. Briefly, the precoated vessels were prepared by incubating them for 1 h at room temperature with 100 µg/ml poly-D-lysine in 10 mM Hepes buffer at 0.165 ml/cm^2^. The vessels were washed three times with water and allowed to completely dry aseptically in the hood. They were then further incubated with 100 µg/ml fibronectin/PBS for 5 min or alternatively 25 µg/ml fibronectin/PBS for 1 h. The fibronectin solution was aspirated and the vessels were used immediately without drying. Approximately 6.1×10^6^ total cells were obtained upon the initial dissociation of the spinal cord tissue. All of the cells were plated onto one 150 mm plate in 20 ml of the growth media.

The growth medium was changed every other day and in the alternate days, 10 ng/ml of bFGF was directly added to the culture. The first passage was conducted 16 days after plating. At this point, the culture was composed mostly of post-mitotic neurons and mitotic hNSCs. The mitotic cells were harvested by brief treatment with trypsin (0.05% in 0.53 mM EDTA). Trypsin was stopped by addition of soybean trypsin inhibitor to 0.05% final concentration. The cell suspension was triturated with a pipette to obtain a single cell suspension and centrifuged at 1400 rpm for 5 min. The cell pellet was resuspended in growth media and the cells were replated in new pre-coated plates at 1.2×10^6^ cells in 20 ml of growth media per 150 mm plate. The cells were harvested at approximately 75% confluence, which occurred in 5–6 days. This process was repeated for 20 passages. At various passages, the cells were frozen in the growth medium plus 10% DMSO at 5×10^6^–10×10^6^ cells/ml using a programmable freezer. The frozen cells were stored in liquid nitrogen. Upon thawing, the overall viability and recovery was typically 80–95%. The resulting cell line, which was produced by epigenetic means only, using bFGF as the sole mitogen, was named “566RSC.” A cell bank of passage 16 cells was prepared and used for this study.

### Preparation of hNSCs for implantation

One day prior to each surgery day, one cryopreserved vial of the previously prepared passage 16 cell bank was thawed, washed, concentrated in a hibernation buffer, and shipped from the cell preparation site (Neuralstem, Inc., Rockville, MD, USA) to the surgery site (UCSD, San Diego, CA, USA) at 2–8°C by overnight delivery. Upon receipt the following day, the cells were used directly for implantation without further manipulation. Before and after implantation the viability of cells was measured with Trypan blue (0.4%; Sigma). On average a 75–85% viability rate was recorded.

### Experimental groups

Before cell grafting, SOD1^G93A^ transgenic rats were randomly divided into 3 experimental groups – no treatment (untreated SOD1^G93A^), media-treated SOD1^G93A^, and cell-grafted SOD1^G93A^; non-transgenic littermates were used as control animals and received no treatment. A total of 24 animals (12 males, 12 females) were assigned to the cell-grafted SOD1^G93A^ group, while 16 animals (8 males, 8 females) were assigned to receive media only. Immunosuppressive treatment with Prograf (FK506; 3 mg/kg/day subcutaneous; Astellas Pharma, Deerfield, IL) and Cellcept (mycophenolate mofetil; 30 mg/kg/day intraperitoneal; Roche Pharmaceuticals, Nutley NJ) were initiated 2 d before transplantation. Cellcept continued to 7 d post-surgery, while Prograf was changed to 1 mg/kg/day at 14 d post-op and continued until the study end. A separate group of athymic rats (Crl:NIH-Foxn1^rnu^; n = 12; Charles River) was used for a subset of experiments but underwent no immunosuppression.

Motor-evoked potentials were recorded from the dorsal surface of the lumbar spinal cord (see Motor-evoked potentials recording) in a separate group of endstage SOD1^G93A^ animals (n = 6) and non-transgenic controls (n = 6) which did not undergo any treatment or spinal injections. The same animals were then used for qualitative and quantitative analysis of axonal degeneration/loss of spinal descending motor tracts using silver impregnation techniques and plastic-embedded semithin sections (see Plastic embedding and Silver degeneration staining).

### Spinal cord implantation of hNSCs

Animals (60–65 d old) were anesthetized with isoflurane (1.5–2% maintenance; in room air), placed into a spinal clamp apparatus (Stoelting, Wood Dale, IL, USA) and a partial T12–L1 laminectomy was performed using a dental drill (exposing the dorsal surface of L2–L6 segments). Using a glass capillary (tip diameter 80–100 µm) connected to a microinjector (Kopf Instruments, Tujunga, CA), rats were injected with 0.5 µl (10,000 cells per injection) of the hNSCs in hibernation buffer, or only the hibernation buffer as control (media). The duration of each injection was 60 s followed by 30 s pause before capillary withdrawal. The center of the injection was targeted into the intermediate zone and ventral horn (distance from the dorsal surface of the spinal cord at L3 level: 1.1–1.2 mm), [47]. Injections were made every 700–900 µm, rostro-caudally, on each side of the lumbar spinal cord targeting L2–L5 segments. The total number of injections ranged between 9–13 injections per side. After injections, the incision was cleaned with penicillin-streptomycin solution and sutured in two layers. Athymic rats were transplanted in an identical fashion.

### Assessment of neurological function and disease progression

*Motor function* was evaluated using the 21-point open field BBB locomotor scale [48]. Animals were observed for 4–5 min and scored by an experimenter blinded to the treatment groups. *Disease onset* was defined as the age of maximum body weight; *Early disease* was defined as the point at which the animal lost 10% of peak body weight; *Endstage* was defined as when an animal could not right itself after 30 s of being placed on its side [38].

### Hoffman reflex (H-reflex) recording

H-reflex was recorded as previously described [49], [50]. Under ketamine anesthesia (100 mg/kg/hr, i.m.) the right hind limb of the animal was secured and a pair of stimulating needle electrodes was transcutaneously inserted into the surroundings of the tibial nerve. For recording a pair of silver needle electrodes was placed into the interosseous muscles between the fourth and the fifth or the first and the second metatarsal right foot muscles. The tibial nerve was stimulated using square pulses with increasing stimulus intensity (0.1–10 mA in 0.5 mA increments, 0.1 Hz, 0.2 ms; WPI; Isostim A320) and responses were recorded with an A/C-coupled differential amplifier (Model DB4; DPI, Sarasota, FL).

### Motor-evoked potential recording

Under isoflurane anesthesia (1.5–2% maintenance; in room air), terminal SOD1^G93A^ rats (n = 6 or age-matched controls, n = 6) without any previous treatment (i.e., no spinal injections) were mounted into a stereotaxic frame and the scalp over motor cortex was cut open to expose the skull. Stimulation was done using a pair of stimulating electrodes consisting of a stainless steel screw placed into the skull over the motor cortex and stainless steel needle inserted into the hard palate behind the upper incisors. Previous data show that such placement of stimulating electrodes provides consistent activation of pyramidal and extrapyramidal system (19–21).

A dental drill was used to perform a laminectomy of T11 vertebra exposing the T12 spinal segment. Evoked responses were recorded by a pair of flexible silver ball electrodes placed on the dura surface of the exposed T12 spinal segment. A reference silver-chloride disc electrode was placed subcutaneously on the contralateral side of the recording. After electrode placement, animals were injected with ketamine (100 mg/kg/h, I.M.) and isoflurane anesthesia was discontinued. Stimulation pulses were 0.2 ms long with amplitudes ranging from 0.5 to 8 mA (Isostim A320R, World Precision Instruments, Florida, USA). Signals from recording electrodes were filtered by 10 kHz low pass filter, amplified by differential amplifier (TDT DB4 amplifier with HS4 preamplifier, Tucker-Davis Technologies, Florida, USA), digitized in 30 kHz sampling frequency and stored for further analysis.

### Immunohistochemistry

At endstage disease, rats were deeply anesthetized with pentobarbital and phenytoin and transcardially perfused with 200 ml of heparinized saline followed by 250 ml of 4% paraformaldehyde in PBS. The spinal cords were dissected and postfixed in 4% formaldehyde in PBS overnight at 4°C and then cryoprotected in 30% sucrose PBS until transverse sections (30 µm thick) were cut on a cryostat and stored in PBS. Sections for brightfield immunohistochemical staining were pretreated with 3% H_2_O_2_ in PBS for 15 min, washed 3× in PBS and were then placed in primary antibody similar as sections for fluorescent staining: overnight at 4°C with primary human specific (h) or non-specific antibodies made in PBS with 0.2% Triton-X100: mouse anti-human nuclear matrix protein/h-nuc (hNUMA; 1∶100; Millipore, Temecula, CA, USA); goat anti-doublecortin (DCX; 1∶1000; Millipore, Temecula, CA); mouse anti-human neuron specific enolase (hNSE; 1∶200, Vector Laboratories Inc., Burlingame, CA); rabbit anti-glial fabrillary acidic protein (GFAP, 1∶1000, Sigma-Aldrich Corp. St. Louis, MO); goat anti-choline acetyltransferase (ChAT; 1∶100; Millipore, Temecula, CA); biotinylated mouse anti-NeuN (Millipore, Temecula, CA); mouse anti-adenomatus polyposis coli (APC; 1∶500; EMD Chemicals Inc., Gibbstown, NJ); chicken anti-beta III tubulin (TUJ1; 1∶1000; Aves Labs Inc, Tigard, OR); rabbit anti-ionized calcium binding adaptor molecule 1 (Iba1; 1∶1000; Wako Chemicals USA, Inc., Richmond, VA); mouse anti-human synaptophysin (hSYN; 1∶1000; Millipore, Temecula, CA); guinea pig anti-vesicular glutamate transporter (VGluT1- 1∶2500; VGluT2 – 1∶2500; VGluT3 – 1∶5000; Millipore, Temecula, CA); guinea pig anti-glycine transporter 2 (GlyT2; 1∶2000; Millipore, Temecula, CA); rabbit anti-growth associated protein 43 (GAP43; 1∶500; Millipore, Temecula, CA); rabbit anti-glutamate decarboxylase 65 (GAD65; 1∶2000; Millipore, Temecula, CA).

After incubation with primary antibodies, sections were washed 3× in PBS and incubated with fluorescent-conjugated secondary antibodies raised in donkey (Alexa 488, 546; 647; 1∶250; Invitrogen Corp., Carlsbad, CA, USA) and DAPI for general nuclear staining. In cases where 2 mouse antibodies were required for multi-labeling, one was biotinylated using a Zenon mouse IgG labeling kit according to the manufacturer's instructions (Invitrogen Corp., Carlsbad, CA, USA). Once staining was complete, sections were mounted on slides, dried at room temperature and covered with Prolong anti-fade kit (Invitrogen Corp., Carlsbad, CA, USA). Sections for brightfield were washed after primary antibody, placed in biotinylated secondary antibody (goat anti-mouse; 1∶500; Vector Laboratories, Burlingame, CA) for 2 h at room temperature. Those sections were again washed, placed in avidin-biotin complex (ABC kit, Vector Laboratories, Burlingame, CA) for 2 h at room temperature. Finally, the sections were washed, developed with 3,3′-diaminobenzidine (DAB; Vector Laboratories, Burlingame, CA), mounted on silane-coated slides, air-dried, dehydrated and coverslipped. Images were captured using a Leica DMLB microscope with a Zeiss Axiocam MRm monochrome camera. Some images were captured using a Leica SP2 confocal microscope. Any image post-processing was done with Adobe CS3 (Adobe Systems, Inc., San Jose, CA) with equal changes to any images being compared.

### Plastic embedding

For plastic embedding, spinal cord blocks (2–3 mm thick) were postfixed in 0.3% glutaraldehyde for 1 day at 4°C. Tissue was rinsed 3×5 min in 0.1 M phosphate buffer (pH 7.4), and stored in phosphate buffer overnight at 4°C. Secondary postfixation was performed using 0.1% osmium tetroxide in 0.1 M phosphate buffer for 12 hours, followed by rinsing in phosphate buffer. This was followed by progressive alcohol dehydration according to standard procedures up to 100% ethanol, with the addition of further dehydration in a 1∶1 solution of ethanol/propylene oxide, and lastly 100% propylene oxide. Dehydrated blocks were then prepared for resin infiltration by incubation in a 1∶1 solution of resin/propylene oxide overnight. The resin included: Eponate 12, Araldite 502, Dodecenyl Succinic Anhydride (DDSA), and 2,4,6-Tri[Dimethylaminomethyl]phenol (DMP-30) (Ted Pella Inc), mixed in ratios of 10∶10∶25∶1 respectively. Blocks were then transferred to 100% resin for subsequent overnight infiltration on a rotator. Finally, tissue blocks were embedded using fresh resin in multi-chamber silicone rubber molds (Ted Pella). The mold was placed in an oven (60°C) for 2 days to facilitate resin polymerization. Semithin (1 µm) transverse sections were then cut using a Leica (DM-40) microtome with glass knives. Sections were mounted to slides from distilled water and allowed to dry on slide warmer. Prior to staining, slides were incubated at 60°C in an oven for 10–15 minutes, and then contrast-stained with 4% para-phenylenediamine (PPD).

### Silver degeneration staining

#### De Olmos aminocupric silver impregnation

Neurodegeneration was assessed using the de Olmos aminocupric silver histochemical technique as previously described [23], [25]. Following the vendor's instructions (Neuroscience Associates, TN, USA), SOD1^G93A^ endstage rats and non-transgenic age-matched littermates were transcardially perfused with cacodylate-modified PBS followed by cacodylate-modified paraformaldehyde and brain and spinal cord tissue shipped to Neuroscience Associates for processing.

#### Gallyas silver impregnation [22]

Paraformaldehyde-fixed spinal cord tissue was embedded in paraffin wax and 12 µm thick sections were cut and mounted directly on slide. After dewaxing in xylene and progressive rehydration to water, the slides were placed in fresh 0.3% magnesium permanganate and subsequently rinsed in tap water for 10 minutes. Further magnesium permanganate clearing was done in 2% oxalic acid for 3 min. Background suppression was facilitated by incubation in fresh 0.004% lanthanum nitrate/0.02% sodium acetate solution for 60 minutes. Following wash, slides were placed into a silver iodide solution for 2 minutes, and then neutralized by three washes in 0.5% acetic acid. The intensity of silver impregnated structures was amplified with by immersion in a 1∶1 ratio of two physical developer solutions: A: ammonium nitrate, silver nitrate, tungstosilic acid, and formaldehyde; and B: 5% anhydrous sodium carbonate solution. Slides were then quickly washed in 0.5% acetic acid, and placed into a 0.5% gold chloride solution for 5–10 minutes. Slides were rinsed in distilled water, placed in 2% sodium sulfate (2 minutes) and rewashed in distilled water before they were progressively dehydrated to xylene and cover slipped in DPX.

### Immuno-electron microscopy

Transverse spinal cord sections (50 µm thick) were prepared from lumbar spinal cords of immunodeficient rats at 9 months after hNSCs grafting. Sections were cut on a vibratome and cryoprotected with glycerol–dimethylsulfoxide mixture. After cryoprotection, the sections were frozen and thawed four times and treated with 1% sodium borohydrate. To reduce nonspecific binding, the sections were treated with 0.3% H_2_O_2_–10% methanol in TBS (100 mM Tris-HCl and 150 mM NaCl, pH 7.6) and 3% NGS–1% bovine serum albumin in TBS. Sections were reacted overnight with mouse anti-human-specific synaptophysin (1∶1000; Chemicon). Bound antibody was detected using biotinylated donkey anti-mouse IgG (1∶500; GE Healthcare, Little Chalfont, UK), the ABC Elite kit (Vector Laboratories, Burlingame, CA), and diaminobenzidine (DAB) as the chromogen. After DAB detection, some sections were processed by an additional antibody labeling cycle using the same method and antibody as above. This staining strategy enhanced the signal-to-background ratio while the background labeling was kept to minimal. Immunoreacted sections were postfixed in buffered 2% OsO_4_, rinsed and stained in 1% uranyl acetate, and then dehydrated and embedded in Epon. Ultrathin sections were contrasted with uranyl acetate and analyzed under a Zeiss EM-10 electron microscope operated at 60–80 kV. Electron microscopic negatives were scanned and processed by Adobe Photoshop CS2 (Adobe Systems).

### Quantitative immunohistochemistry

#### GFAP/Iba1 densitometry

Immunohistochemical staining was performed using the same antibody solution for all free-floating sections and processed as described above. Randomly selected four sections (from L4 and L5 spinal cord segments) were analyzed from each animal. Using identical camera settings, images were obtained from lamina IX on both sides of each section. Using the pixel histogram generated for each original image by Image-Pro Plus (v.6.2.0.424; Media Cybernetics Inc., Bethesda, MD), the product of the pixel number and pixel intensity value (0–255) was computed and summed for the entire image. This provides a composite measure of changes in both immunoreactive area and intensity.

#### Neuronal bouton analyses

Bouton analyses were performed as previously described, [51]–[54]. The investigator was blinded during analyses. Using immunofluorescence-stained sections and identical microscope settings, 3 images (75 µm×75 µm) were captured from lamina IX (adjacent to a CHAT+ α-motoneuron surrounded by hSYN staining) of each section using a Leica SP2 confocal microscope; at least 3 sections (300 µm separation) were used from each of 5 animals. Image-Pro Plus (v.6.2.0.424; Media Cybernetics Inc., Bethesda, MD) was used to count the total number of human synaptophysin-immunoreactive objects using the same limits for pixel intensity and structure size. The image was then overlaid onto another image from the identical field but stained for other neuronal phenotype markers (VGluT1/2/3, GAD65, GlyT2). The total number of double-positive structures was then identified by Image-Pro Plus. The number of hSYN+ punctata identified on each α-motoneuron ranged between 30–80 per α-motoneuron.

#### α-motoneuron quantification

Alpha-motoneuron quantification was performed as previously described [55] except using fluorescent-stained (ChAT) tissue sections. The investigator was blinded during all analyses. Five sections taken from C5–C6 or L4–L5 segments, each separated by minimum of 300 µm, were selected from each animal (n = 18 for the cell-grafted group and n = 16 for the media-injected group; i.e., a total of 90 and 80 sections, respectively) and were identically immunostained for ChAT. Sections from grafted animals had graft presence confirmed by additional hNUMA staining. A single 20× image was then captured (focused in the center of the section thickness) from each lamina IX and analyzed with Image-Pro Plus (v.6.2.0.424; Media Cybernetics Inc., Bethesda, MD) to determine CHAT+ α-motoneurons with surface area >700 µm and a staining intensity that was at least 2-fold over background (same threshold used for all α-motoneuron analyses) [56]. Cells identified by Image-Pro Plus were then examined and those with distinct nucleoli, as defined by clearly detectable lack of CHAT immunoreactivity in the nucleoli, were counted (see **Fig. S 4C** for details).

#### Graft phenotype characterization (NeuN, GFAP, APC, DCX)

To determine the phenotype of the grafted human cells, tissue sections from the L4–L5 engrafted region were double-stained with hNUMA and either NeuN, GFAP, APC, or DCX, as described above. Using identical microscope settings, a z-stack of optical images (0.5-µm-thick; 20× objective) were captured from identified hNUMA+ grafts using a Leica SP2 confocal microscope; at least 3 sections (300 µm separation) were used from each of the 5 animals. Image-Pro Plus (v.6.2.0.424; Media Cybernetics Inc., Bethesda, MD) was then used to identify positive cells in one overlaid optical image (0.5-µm-thick) using identical thresholds for all images. All images were examined by a blinded observer.

### Axonal Quantification

The total number of remaining axons was determined in the lateral and ventral funiculi using semi-automated image analysis of osmium-treated 1-µm plastic sections taken from the lumbar spinal cord of normal (n = 2) and SOD1^G93A^ (n = 2) endstage animals with no previous manipulations (i.e., no spinal media or cell injections).

High resolution mosaic images were obtained using Zeiss Observer software with Multidimensional Acquisition MosiaX (Z1 microscope system with 20× objective fitted with a Zeiss MRm camera, AxioVision v4.7). Using the same pixel threshold to identify axons in all images, Image-Pro Plus (v.6.2.0.424; Media Cybernetics Inc., Bethesda, MD) was used to objectively count axons. Further measurement parameters such as area/box and size (length) were applied to discriminate and exclude non-axonal objects. Employment of the size (length) parameter allowed for further axonal analysis in which axons were divided into empirically derived caliber sizes of small, medium, and large axons (0.5–2.0 µm, 2.01–5.0 µm, and 5.01–14.0 µm respectively).

### Statistical analysis

Two-way comparisons were performed by student t-test. Multiple comparisons were performed using one-way analysis of variance (ANOVA) followed by Student-Newman-Keuls test. All results are shown as mean ± standard error of mean (SEM) unless indicated. P<0.05 was considered to be statistically significant.

## Supporting Information

Figure S1 **Grafted human spinal neural stem cells show expression of several neuronal markers.** Histological sections taken from regions containing human spinal neural stem cell transplants were immunostained with neuronal cells markers TUJ1, DCX and GAP43. Human cells were identified by the presence of human-specific nuclear matrix antigen (hNUMA), (A, D). Cell grafts were typically concentrated in the deeper lamina (VII–IX) and frequently extended into the adjacent white matter. Regions stained for hNUMA were also strongly stained for beta-tubulin III (TUJ1; B), doublecortin (DCX; E), and growth-associated protein 43 (GAP43; F). Scale bar (G) is 300 µm for all panels.
(TIF)

Figure S2 **Grafted, terminally differentiated human neural spinal stem cells-derived neurons develop putative synaptic contact with persisting α-motoneurons in SOD1^G93A^ rats.** Human synaptophysin (hSYN) was detected throughout the cell grafts, often found in axonal-like structures with typical varicosities (B insert) and in the vicinity of persisting α-motoneurons (ChAT; choline acetyltranseferase) in lamina IX and extending into the adjacent white matter (A–D). Single optical layer confocal images of surviving α -motoneurons show hSYN-IR bouton-like structures adjacent to the outer membrane of the soma (E, F), occasionally expressing the GABAergic cell marker glutamate decarboxylase (GAD65), (G, H). Human glutamatergic boutons were located by identifying specific glutamate vesicular transporters 1/2/3 (VGluT) and similarly showed only rare boutons also reactive for hSYN (I–L). Glycinergic boutons were identified by the neuronal-specific glycine transporter 2 (GlyT2), (M–P). Arrows show examples of double-immunoreactive structures. Scale bar: 300 µm (A–D), 25 µm (E–P).
(TIF)

Figure S3 **Spinal grafts of human spinal neural stem cells reduced astrogliosis and microglial activation in SOD1^G93A^ rats.** Quantitative densitometry was performed on lumbar (L4 and L5) spinal cord sections immunostained for astrocytes (GFAP) and microglia (Iba1). Lamina IX images were captured from non-transgenic (A–C), untreated SOD1^G93A^ (D–F), media-treated SOD1^G93A^ (G–I), and cell-grafted SOD1^G93A^ (J–L) animals. All three SOD1^G93A^ groups show signs of strong astrogliosis and microglia infiltration/activation, with a marked increase in the number of GFAP-IR hypertrophic astrocytes and dense Iba1-immunoreactivity (IR). Based on densitometric analyses, lamina IX GFAP-IR and Iba1-IR in SOD1^G93A^ and media-treated groups were significantly increased over the non-transgenic group (M). Reduced GFAP-IR and Iba1-IR was measured in the cell-grafted group (M). Scale bar: 80 µm. (* significantly increased over non-transgenic; P<0.05; one-way ANOVA); ** significantly increased over non-transgenic but decreased from media-treated; P<0.05; one-way ANOVA).
(TIF)

Figure S4 **Migration of grafted human fetal spinal neural stem cells in lumbar spinal cord in SOD1^G93A^ rats at 78 days after grafting or in immunodeficient rats at 9 months after grafting.** By comparing the spread of hNUMA+ cells in SOD1^G93A^ rats at 78 days after grafting (A) with that seen in immunodeficient rats at 9 months (B), wide spread of grafted cells in both the gray matter and white matter was seen at 9 months (A, B; compare red asterisks in the dorsal and lateral funiculi). To quantify α-motoneurons, CHAT immunofluorescence-stained sections were used. Cells to be counted were identified by surface area (>700 µm) and by the presence of nucleoli as evidenced by an easily identifiable lack of CHAT staining in the center of nucleus (C; red arrows).
(TIF)
