# Supplementary figures and images for: Human Neural Stem Cell Replacement Therapy for Amyotrophic Lateral Sclerosis by Spinal Transplantation
Source: PLoS One. 2012 Aug 20;7(8):e42614. doi: 10.1371/journal.pone.0042614 (PMC3423406; doi:10.1371/journal.pone.0042614)

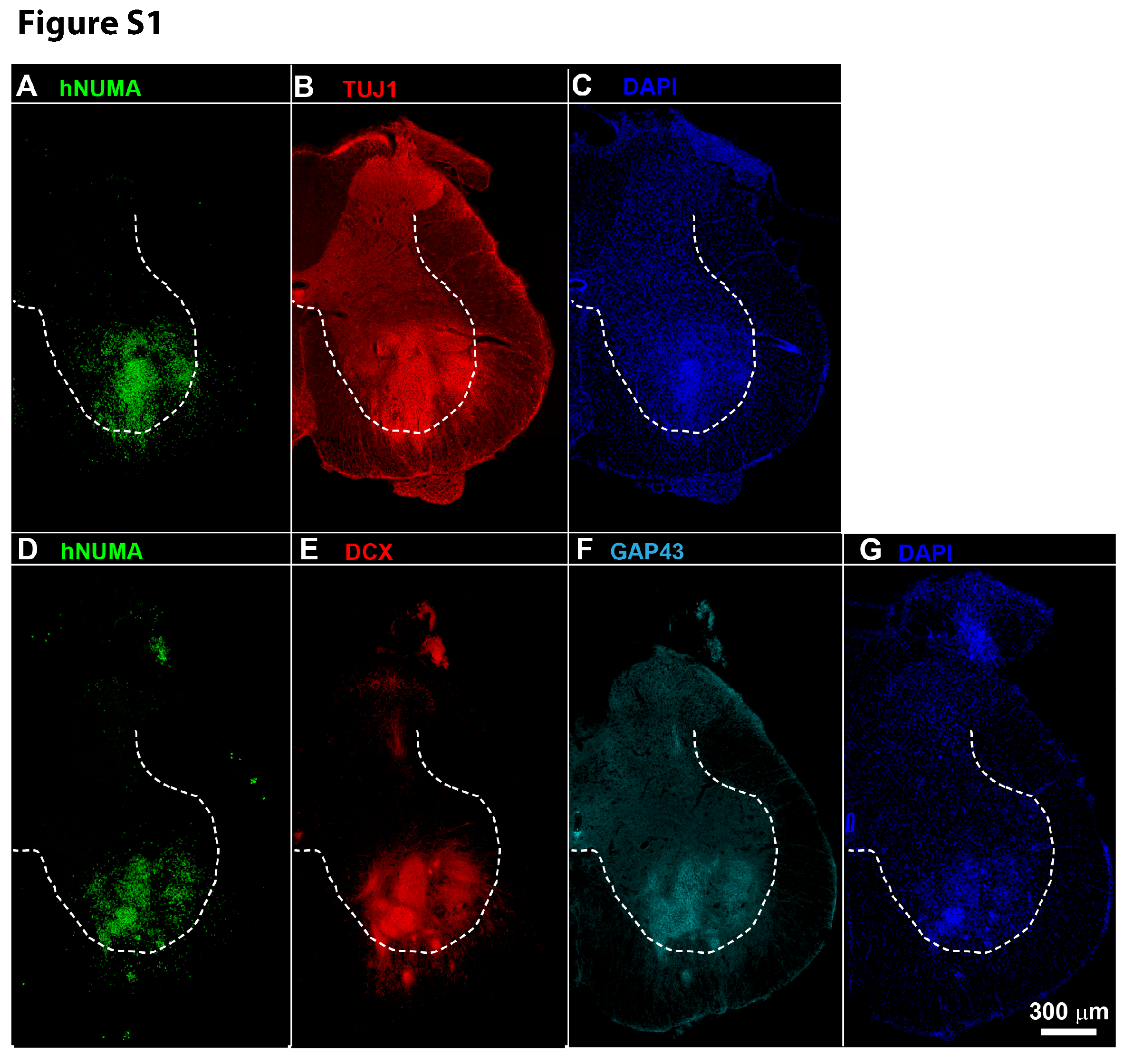

Supplement: Figure S1 — Grafted human spinal neural stem cells show expression of several neuronal markers. Histological sections taken from regions containing human spinal neural stem cell transplants were immunostained with neuronal cells markers TUJ1, DCX and GAP43. Human cells were identified by the presence of human-specific nuclear matrix antigen (hNUMA), (A, D). Cell grafts were typically concentrated in the deeper lamina (VII–IX) and frequently extended into the adjacent white matter. Regions stained for hNUMA were also strongly stained for beta-tubulin III (TUJ1; B), doublecortin (DCX; E), and growth-associated protein 43 (GAP43; F). Scale bar (G) is 300 µm for all panels. (TIF) [file pone.0042614.s001.tif]

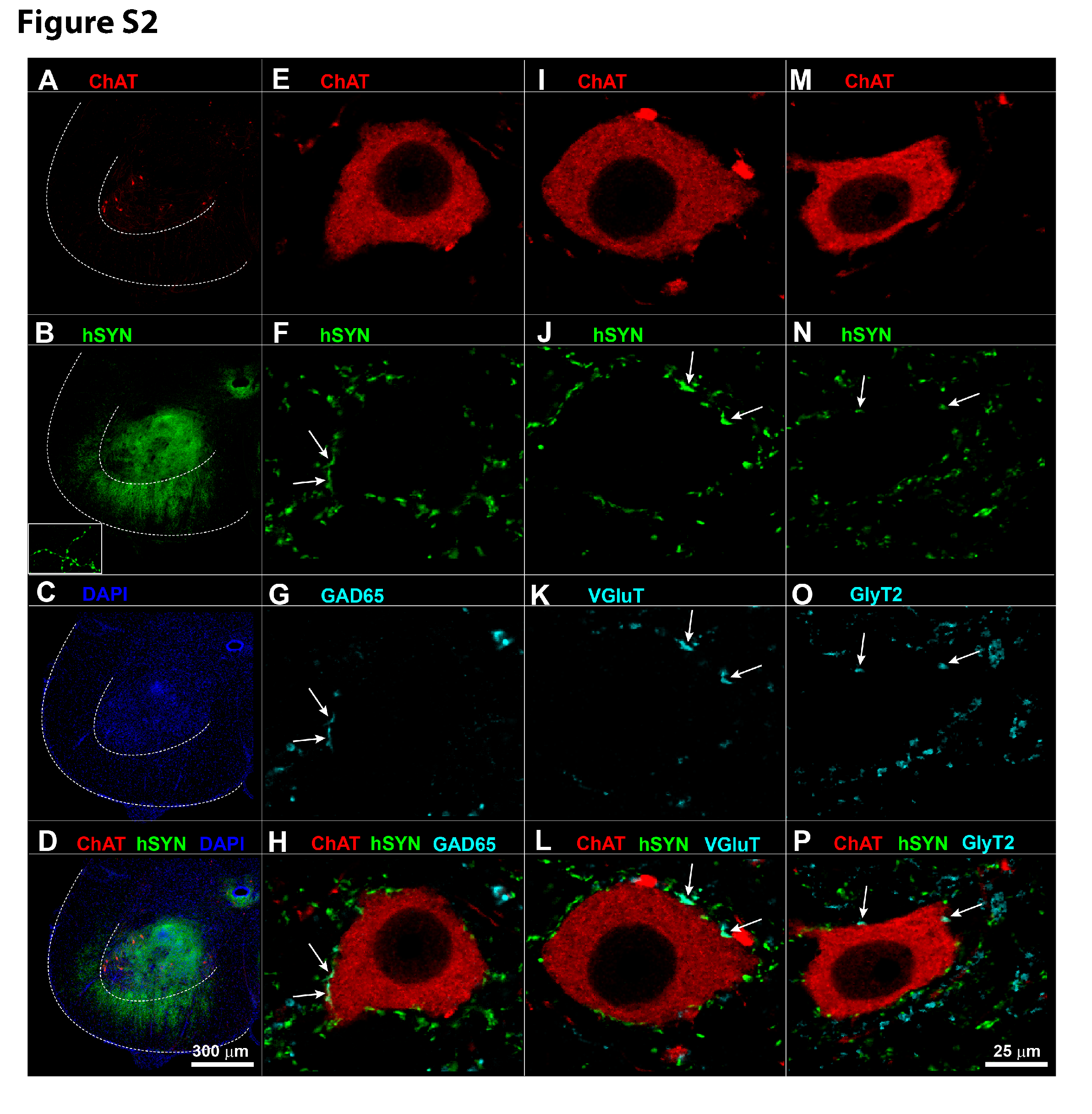

Supplement: Figure S2 — Grafted, terminally differentiated human neural spinal stem cells-derived neurons develop putative synaptic contact with persisting α-motoneurons in SOD1G93A rats. Human synaptophysin (hSYN) was detected throughout the cell grafts, often found in axonal-like structures with typical varicosities (B insert) and in the vicinity of persisting α-motoneurons (ChAT; choline acetyltranseferase) in lamina IX and extending into the adjacent white matter (A–D). Single optical layer confocal images of surviving α -motoneurons show hSYN-IR bouton-like structures adjacent to the outer membrane of the soma (E, F), occasionally expressing the GABAergic cell marker glutamate decarboxylase (GAD65), (G, H). Human glutamatergic boutons were located by identifying specific glutamate vesicular transporters 1/2/3 (VGluT) and similarly showed only rare boutons also reactive for hSYN (I–L). Glycinergic boutons were identified by the neuronal-specific glycine transporter 2 (GlyT2), (M–P). Arrows show examples of double-immunoreactive structures. Scale bar: 300 µm (A–D), 25 µm (E–P). (TIF) [file pone.0042614.s002.tif]

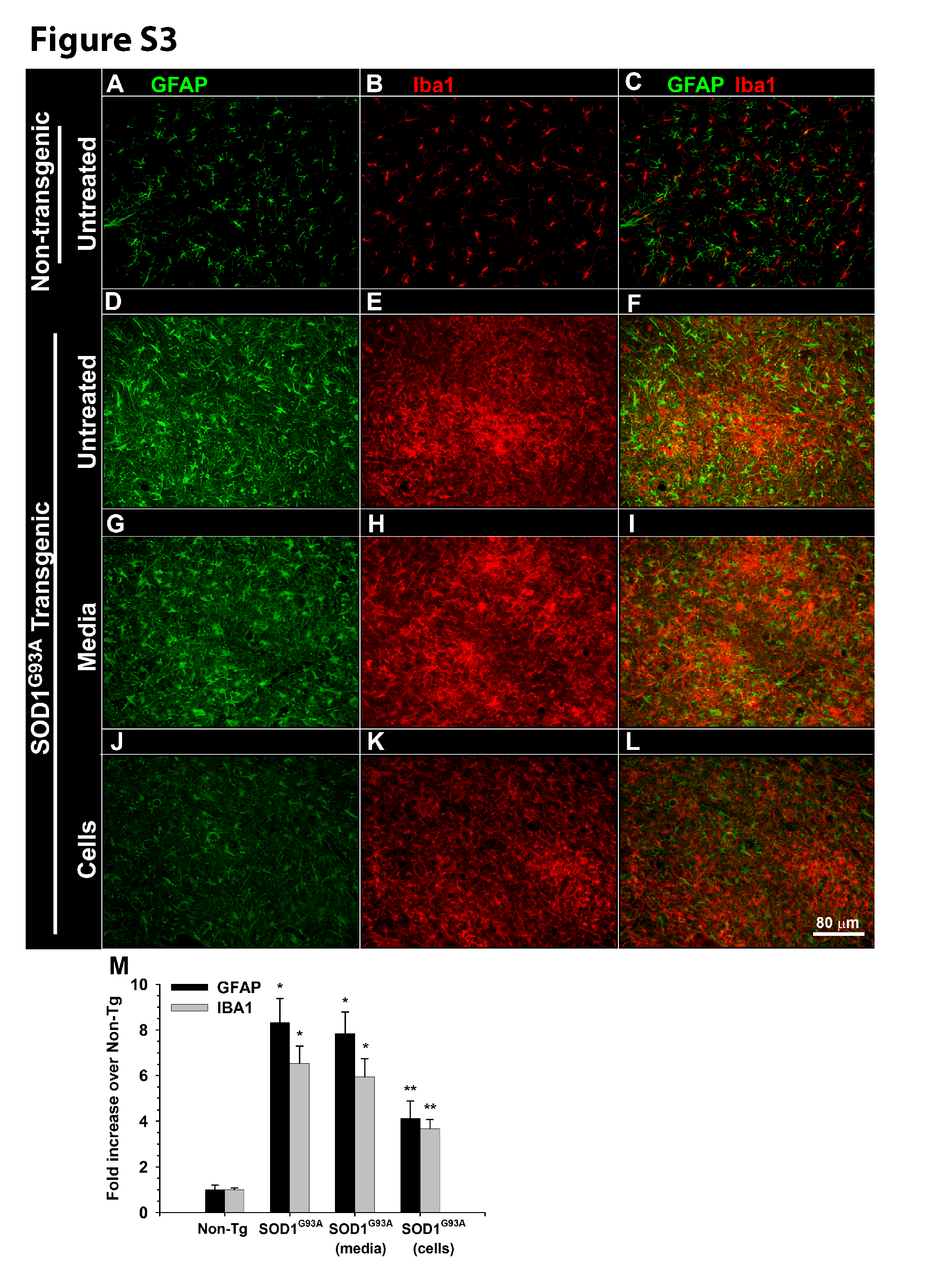

Supplement: Figure S3 — Spinal grafts of human spinal neural stem cells reduced astrogliosis and microglial activation in SOD1G93A rats. Quantitative densitometry was performed on lumbar (L4 and L5) spinal cord sections immunostained for astrocytes (GFAP) and microglia (Iba1). Lamina IX images were captured from non-transgenic (A–C), untreated SOD1G93A (D–F), media-treated SOD1G93A (G–I), and cell-grafted SOD1G93A (J–L) animals. All three SOD1G93A groups show signs of strong astrogliosis and microglia infiltration/activation, with a marked increase in the number of GFAP-IR hypertrophic astrocytes and dense Iba1-immunoreactivity (IR). Based on densitometric analyses, lamina IX GFAP-IR and Iba1-IR in SOD1G93A and media-treated groups were significantly increased over the non-transgenic group (M). Reduced GFAP-IR and Iba1-IR was measured in the cell-grafted group (M). Scale bar: 80 µm. (* significantly increased over non-transgenic; P<0.05; one-way ANOVA); ** significantly increased over non-transgenic but decreased from media-treated; P<0.05; one-way ANOVA). (TIF) [file pone.0042614.s003.tif]

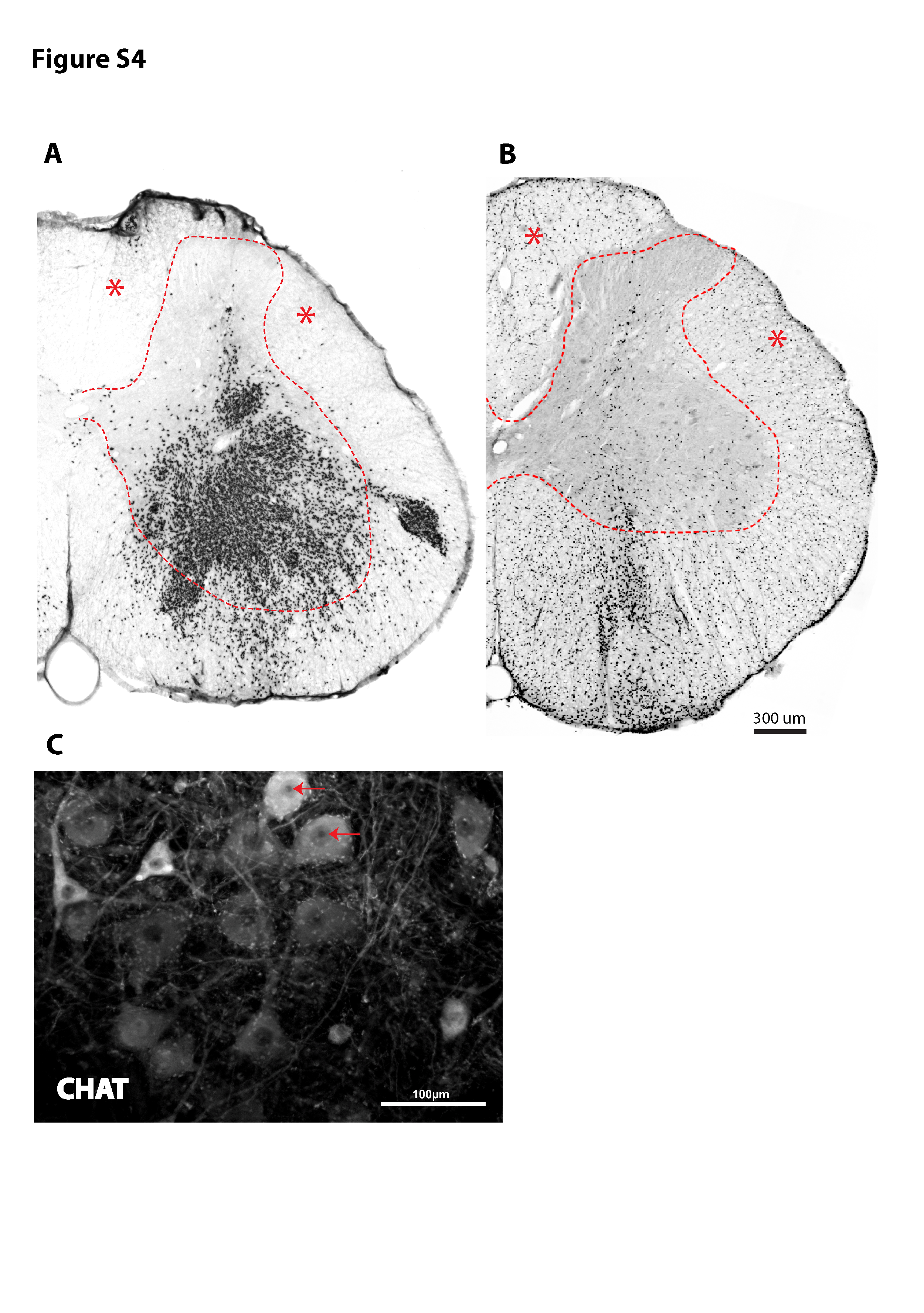

Supplement: Figure S4 — Migration of grafted human fetal spinal neural stem cells in lumbar spinal cord in SOD1G93A rats at 78 days after grafting or in immunodeficient rats at 9 months after grafting. By comparing the spread of hNUMA+ cells in SOD1G93A rats at 78 days after grafting (A) with that seen in immunodeficient rats at 9 months (B), wide spread of grafted cells in both the gray matter and white matter was seen at 9 months (A, B; compare red asterisks in the dorsal and lateral funiculi). To quantify α-motoneurons, CHAT immunofluorescence-stained sections were used. Cells to be counted were identified by surface area (>700 µm) and by the presence of nucleoli as evidenced by an easily identifiable lack of CHAT staining in the center of nucleus (C; red arrows). (TIF) [file pone.0042614.s004.tif]
